# Basic Properties of the p38 Signaling Pathway in Response to Hyperosmotic Shock

**DOI:** 10.1371/journal.pone.0135249

**Published:** 2015-09-03

**Authors:** Nabil Ben Messaoud, Ilina Katzarova, José M. López

**Affiliations:** Institut de Neurociències, Departament de Bioquímica i Biología Molecular, Unitat de Bioquímica, Facultad de Medicina, Universitat Autònoma de Barcelona, 08193 Cerdanyola del Vallès, Barcelona, Spain; The University of Tokyo, JAPAN

## Abstract

Some properties of signaling systems, like ultrasensitivity, hysteresis (a form of biochemical memory), and all-or-none responses at a single cell level, are important to understand the regulation of irreversible processes. *Xenopus* oocytes are a suitable cell model to study these properties. The p38 MAPK (mitogen-activated protein kinase) pathway is activated by different stress stimuli, including osmostress, and regulates multiple biological processes, from immune response to cell cycle. Recently, we have reported that activation of p38 and JNK regulate osmostress-induced apoptosis in *Xenopus* oocytes and that sustained activation of p38 accelerates cytochrome c release and caspase-3 activation. However, the signaling properties of p38 in response to hyperosmotic shock have not been studied. Here we show, using *Xenopus* oocytes as a cell model, that hyperosmotic shock activates the p38 signaling pathway with an ultrasensitive and bimodal response in a time-dependent manner, and with low hysteresis. At a single cell level, p38 activation is not well correlated with cytochrome c release 2 h after hyperosmotic shock, but a good correlation is observed at 4 h after treatment. Interestingly, cytochrome c microinjection induces p38 phosphorylation through caspase-3 activation, and caspase inhibition reduces p38 activation induced by osmostress, indicating that a positive feedback loop is engaged by hyperosmotic shock. To know the properties of the stress protein kinases activated by hyperosmotic shock will facilitate the design of computational models to predict cellular responses in human diseases caused by perturbations in fluid osmolarity.

## Introduction

Stress protein kinases are fundamental for many biological processes mediating the response of the cell to internal or external changes. A cell under stress uses the biological machinery engaging programs to overcome challenging situations. However, if the stress signal persists or became too strong a new program is initiated leading to cell death. The environmental changes that a cell must face are diverse, including alterations in the concentrations of nutrients, growth factors, damaging agents, and changes in the temperature, pH or osmolarity.

The p38 MAPK (mitogen-activated protein kinase) pathway is activated by different stress stimuli and play important roles in the immune and inflammatory response, differentiation, cell cycle and cell survival [1,2]. The first member of the p38 MAPK family was independently identified by four groups [3–6] as a 38 kDa protein (p38) that was rapidly phosphorylated in response to different stimuli, including hyperosmolarity [3]. This protein was found to be the homologue of *Saccharomyces cerevisiae* Hog1, an important regulator of the osmotic response [7]. p38 MAPKs are activated by dual phosphorylation of tyrosine and threonine residues in a conserved Thr-Gly-Tyr motif, in the activation loop, by MKK3 and MKK6 [8–10]. In some circumstances, such as ultraviolet radiation, MKK4, an activator of JNK, may contribute to p38 activation [11].

We have reported that hyperosmotic stress induces apoptosis in *Xenopus* oocytes and activation of the stress protein kinases AMPK (AMP-activated protein kinase) and JNK (c-Jun N-terminal kinase) [12]. By using this cell system we described some basic properties of kinases that are important for the control of irreversible processes: ultrasensitivity (a very large response to a small increase in stimulus after a threshold is crossed), hysteresis (sustained activation when the stimulus has disappeared), and digital (all-or-none) response at a single cell level. We showed that both AMPK and JNK signaling systems were ultrasensitive and digital in response to hyperosmotic shock, and that JNK presented hysteresis whereas AMPK did not [12]. We also proposed a model where the integration of multiple digital signals from stress sensors (protein kinases) would determine the life or death decision in the cell [12,13].

More recently, we have reported that sustained activation of p38 and JNK contribute, in combination with early Smac/DIABLO release and calpain activation, to osmostress-induced apoptosis [14]. However, the signalling properties mentioned before (ultrasensitivity, hysteresis, and analog/digital responses) have not been studied in detail for the p38 pathway. Here we describe these properties in *Xenopus* oocytes exposed to hyperosmotic shock and we discuss their relevance in the control of osmostress-induced apoptosis.

## Materials and Methods

### Oocyte isolation and treatment

Oocytes were obtained from sexually mature *Xenopus laevis* females (purchased from Centre d’Elevage de Xenopes, Montpellier, France). Frogs were kept in aquariums with non chlorinated water at optimum temperature (18°C), with alternating periods of light and darkness (12 h), and fed with a combination of Premium Frog Food (Xenopus Express) and mealworms. Animals were anesthetized in 0.02% benzocaine and portions of ovary were removed through a small incision on the abdomen. The incision was sutured and the animal was returned to a separate tank until it had fully recovered from the anaesthesia. It was then returned to a large tank in which all the frogs were kept for at least 4 weeks until the next surgery. The protocol was approved by the Committee on the Ethics of Animal Experiments of the Universitat Autònoma de Barcelona (Permit Number: CEEAH 439) and all efforts were made to minimize animal suffering. The tissue was examined to ensure the ovaries were healthy and dissected in small pieces. Oocytes were defolliculated for 2–3 h at room temperature with collagenase/dispase (0.8 mg/ml (Sigma), 0.48 mg/ml (Roche)) in MBS (5 mM HEPES, 88 mM NaCl, 1 mM KCl, 1 mM MgSO_4_·7H2O, 2.5 mM NaHCO_3_, 0.7 mM CaCl_2_, pH 7.8) with agitation. The oocytes were then washed thoroughly with MBS and transferred to petri dishes. Stage VI oocytes were sorted manually and incubated overnight at 18°C in MBS supplemented with 0.1 mg/ml gentamicin (Sigma) and 0.05 mg/ml tetracycline (Sigma) to prevent bacterial growth. The next day, healthy survivors were selected and transferred to a petri dish containing fresh MBS with antibiotics. Oocytes were exposed to hyperosmotic shock by transferring them to a new dish containing MBS with sorbitol at different concentrations, collected at different times, and treated as described below. Pools of oocytes were treated with drugs at the concentrations and times indicated, or injected with cytochrome c, and processed as described below.

### Oocyte lysis and Western blot analysis

Fresh oocytes were lysed by pippeting up and down in 200 μl (pools of 20 oocytes) of ice-cold extraction buffer (0.25 M sucrose, 0.1 M NaCl, 2.5 mM MgCl_2_, 20 mM HEPES, pH 7.2) containing 1 mM EDTA, 1 mM EGTA, protease inhibitors (10 μg/ml leupeptin, 1 mM PMSF, 10 μg/ml aprotinin) and phosphatase inhibitors (50 mM β-glycerolphosphate, 50 mM sodium fluoride, 1 mM sodium orthovanadate, 5 mM sodium pyrophosphate). Samples were clarified by centrifugation at 14,500 rpm for 5 min and supernatants were collected and processed for immunoblotting or caspase assay as described below. The whole supernatants were denatured with Sample Buffer (50 mM Tris HCl, pH 6.8, SDS 2%, 100 mM dithiothreitol, 10% glycerol) and subjected to 10% or 15% SDS/PAGE and transferred to Immobilon-P membranes (Millipore). Uniformity of samples loading was verified by Ponceau (Sigma) staining of the blots. Membranes were blocked for 1 h with 5% dried skimmed milk in TBST (50 mM Tris, 150 mM NaCl, 100 mM KCl, pH 7.4, and 0.1% Tween 20) and then incubated with the following antibodies: anti-AMPKα (#2532, Cell Signaling), anti-pp38 (Thr180/Tyr182) (#9211, Cell Signaling), anti-p38 (sc-7149, Santa Cruz), and anti-Cytochrome c (556432, BD Pharmingen). Antibody binding was detected with horseradish peroxidase–coupled secondary antibody and the enhanced chemiluminescence (ECL) detection kit (Amersham).

### Assay for DEVDase activity

Caspase-3 activity was measured in terms of assayed DEVDase activity. From each cytosolic fraction 25 μl (corresponding to 2.5 oocytes) were assayed for DEVDase activity using the synthetic peptide Z-DEVD-AMC from EnzChek Caspase-3 Assay Kit (Molecular Probes). Fluorescence at 360 nm for excitation and at 460 nm for emission was measured after incubation for 1 h at 37°C.

### Oocyte injection

Stage VI *Xenopus* oocytes were injected near their equator with cytochrome c from horse heart (C-7752, Sigma), cytochrome c from Saccharomyces cerevisiae (C2436, Sigma), or bovine serum albumin (BSA) (A9647, Sigma), and maintained in MBS. Pools of 20 oocytes were collected for the indicated times and analyzed by Western blot and caspase-3 activity as previously described. Some oocytes were injected with horse heart cytochrome c plus caspase inhibitors or with DMSO as a control, maintained in new medium and collected and processed as reported before.

### Single oocyte analysis

*Xenopus* oocytes are appropriate for biochemical determinations at a single cell level, since the protein content of one oocyte is equivalent to 250,000 typical somatic cells. Single oocytes were obtained and exposed to hyperosmotic shock following the same procedures that for pools of oocytes. For Western blot analysis each oocyte was lysed in 16 μl of ice-cold extraction buffer with protease and phosphatase inhibitors (instead of the 200 μl used for pools of 20 oocytes). Each sample was clarified by centrifugation at 14,500 rpm for 5 min and the whole supernatant was collected and processed for immunoblotting assay as described before. Therefore, for each oocyte only one Western blot was performed, and when pp38 and p38 levels were determined in the same sample stripping and reproving of the membranes was necessary.

### Statistical analysis

Data are expressed as means ± SEM. ANOVA followed by Dunnett’s test was used to compare caspase-3 activity in oocytes exposed to sorbitol with non treated oocytes. Values of p<0.05 were considered to be statistically significant. The correlation between pp38 and cytochrome c (CC) in single oocytes was performed with the data obtained after quantitation of pp38 and CC levels by Western blot. Calculation of correlation coefficients was performed with GraphPad Prism4. Both Pearson Test (assume a Gaussian distribution) and Spearman test (no assumption about the distribution of the values) were calculated considering two-tailed P value and 95% confidence intervals.

## Results

### Hyperosmotic stress induces an ultrasensitive and monostable response of p38

**Fig 1:**
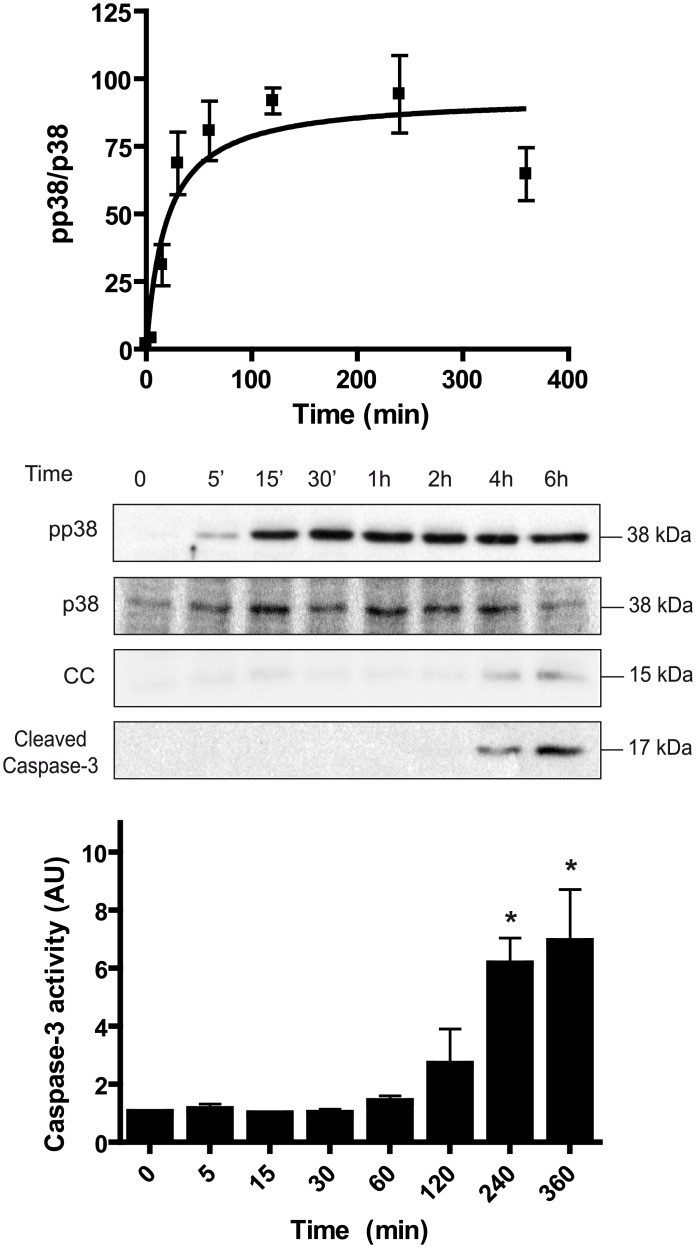
Hyperosmotic shock activates p38 in *Xenopus laevis* oocytes. Oocytes (stage VI) were treated with sorbitol (400 mM) and pools of 20 oocytes were lysed at different times to analyze pp38, p38, cytochrome c (CC), and cleaved caspase-3 by Western blot. p38 activity is represented as pp38/p38 ratio, giving 100% value to the highest activity. Results are represented as mean ± SEM of four independent experiments. A representative Western blot is shown. Caspase-3 activity was determined at different times, using the synthetic peptide Z-DEVD-AMC as a substrate and giving value 1 to non treated oocytes. Data are represented as mean ± SEM (n = 3), *p<0.05 (ANOVA and Dunnett’s test) comparing with non treated oocytes.

**Fig 2:**
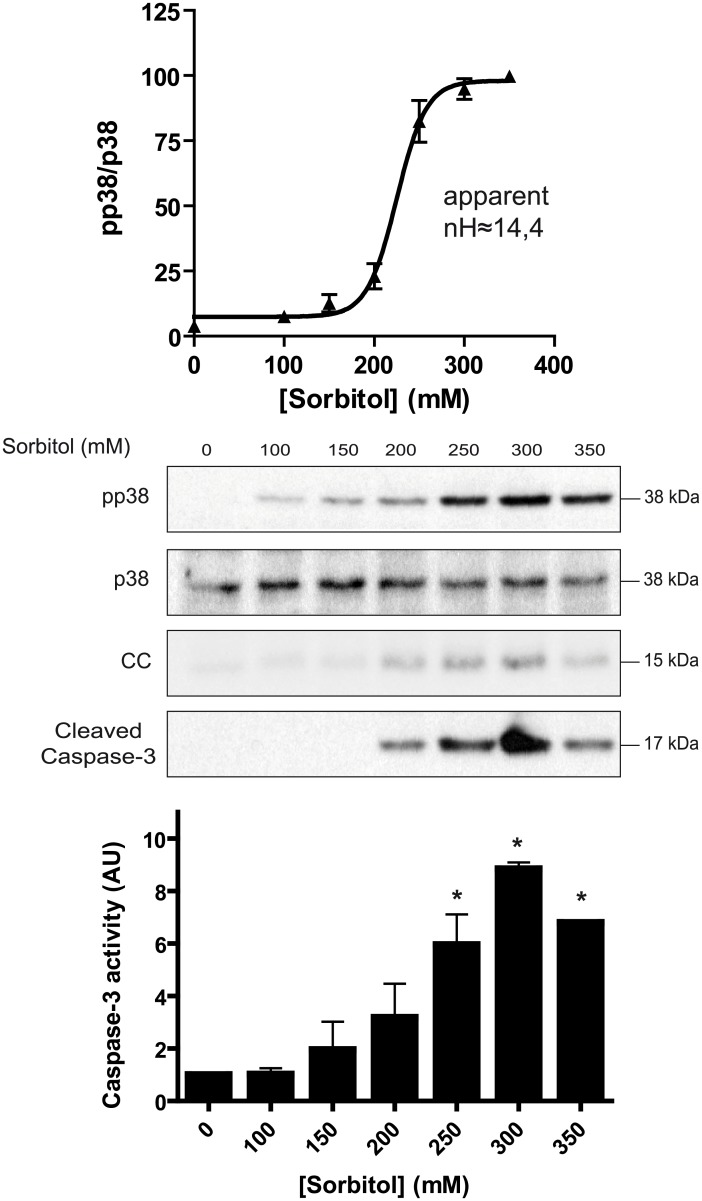
p38 is ultrasensitive to hyperosmolar sorbitol. Oocytes were treated with increasing concentrations of sorbitol for 4 h and pools of 20 oocytes were collected and lysed to analyze pp38, p38, cytochrome c (CC), and cleaved caspase-3 by Western blot. p38 activity is represented as pp38/p38 ratio, giving 100% value to the highest activity. Results are represented as mean ± SEM of four independent experiments. Hill coefficient (nH) was calculated with SAS 9.2 informatics program and represented with GraphPad Prism 4 program. A representative Western blot is shown. Caspase-3 activity was determined at different concentrations of sorbitol as reported before. Data are represented as mean ± SEM (n = 3), *p<0.05 (ANOVA and Dunnett’s test) comparing with non treated oocytes.

**Fig 3:**
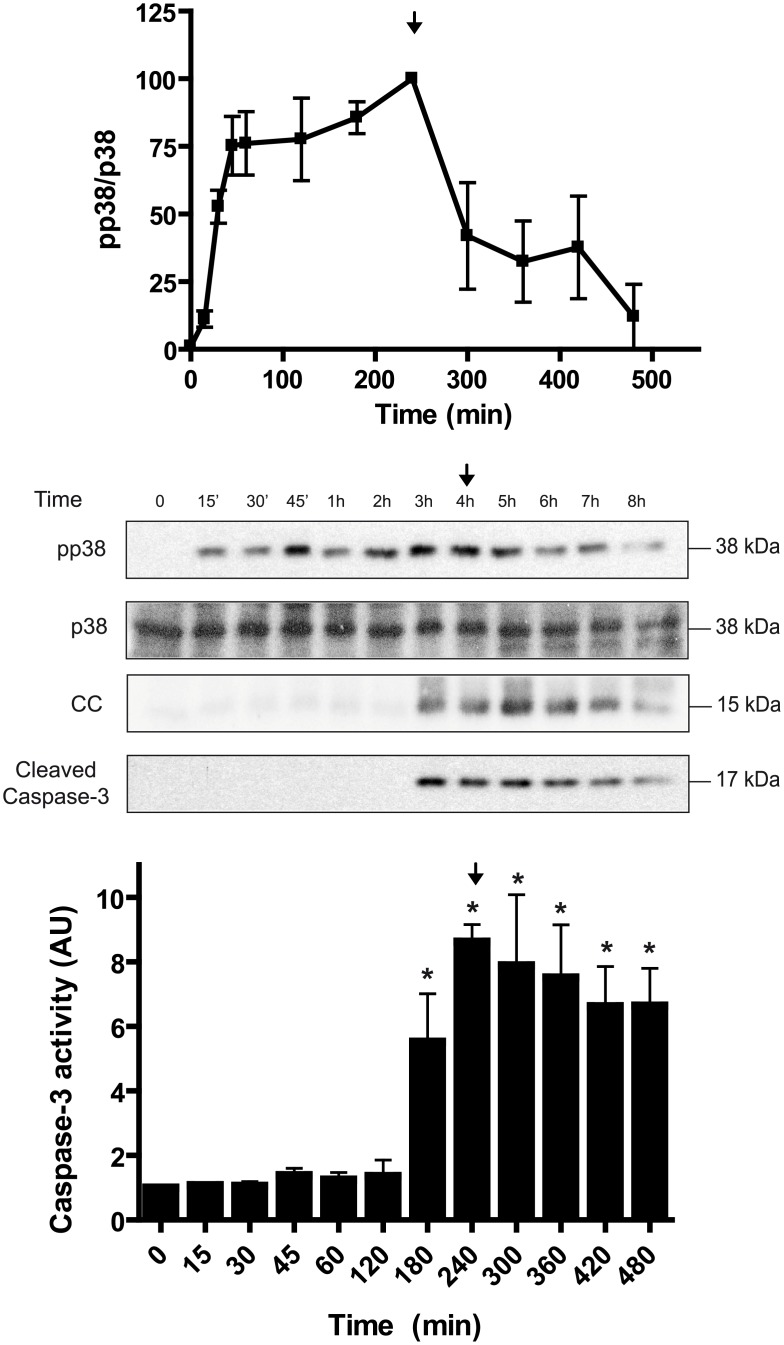
Monostability in the p38 signaling system. Time course of p38 activation in oocytes treated with 300 mM sorbitol. After 4 h of treatment (arrow), oocytes were washed several times with MBS and maintained in MBS without sorbitol. Pools of 20 oocytes were collected at different times to determine pp38, p38, cytochrome c (CC), and cleaved caspase-3 by Western blot. p38 activity is represented as pp38/p38 ratio, given 100% value to the highest activity. Results are represented as mean ± SEM of three independent experiments. A representative Western blot is shown. Caspase-3 activity was determined as reported before. Data are represented as mean ± SEM (n = 3), *p<0.05 (ANOVA and Dunnett’s test) comparing with non treated oocytes.

Osmotic stress activates the AMPK, JNK, and p38 signaling pathways in *Xenopus* oocytes [12,14,15]. As shown in Fig 1, hyperosmolar sorbitol (400 mM) induced a rapid phosphorylation of p38 at Thr181 and Tyr183 in *Xenopus* oocytes. The overall activity of p38 is well correlated with the dual phosphorylation at these residues [16]. Therefore, we quantified the ratio pp38/p38 and referred this value as p38 activity. The time-course experiment showed that hyperosmolar sorbitol increased p38 activity to plateau within 1 h, remaining high through at least 6 h (Fig 1). Cytochrome c release and caspase-3 activation, measured as cleaved caspase-3 by Western blot, were increased at 4 h after treatment (Fig 1). Accordingly, caspase-3 activity, measured in terms of DEVDase activity, was significantly increased at 4 h after treatment (Fig 1). Next, we performed a dose-response experiment at 4 h, a clear steady-state situation. p38 showed a very high ultrasensitivity to increasing concentrations of sorbitol (100–350 mM), with an apparent Hill coefficient of 14.4 (Fig 2). As we had previously reported, the JNK and AMPK signaling systems are also ultrasensitive to hyperosmolar sorbitol with Hill coefficients of 8.8 and 5.3, respectively [12]. Significant levels of cytochrome c (CC), cleaved caspase-3, and caspase-3 activity were observed in oocytes exposed to sorbitol at concentrations ranging from 200 to 350 mM (Fig 2). When we examined the reversibility of the p38 response in pools of oocytes incubated with 300 mM sorbitol during 4 h and washed and returned to normal buffer, we found that p38 activity almost returned to basal levels 4 h after washing (Fig 3). Therefore, p38 is an ultrasensitive monostable system in response to osmostress, similar to AMPK and in contrast to the bistable response of JNK previously reported [12,17]. Note, however, that low levels of p38 activity are still present at 4 h after washing. A significant increase of cytochrome c release and caspase-3 activity was observed at 3 h after treatment, and their levels remained high during the 4 h after washing (Fig 3).

### Bimodal response of p38 to hyperosmotic shock in individual oocytes

**Fig 4:**
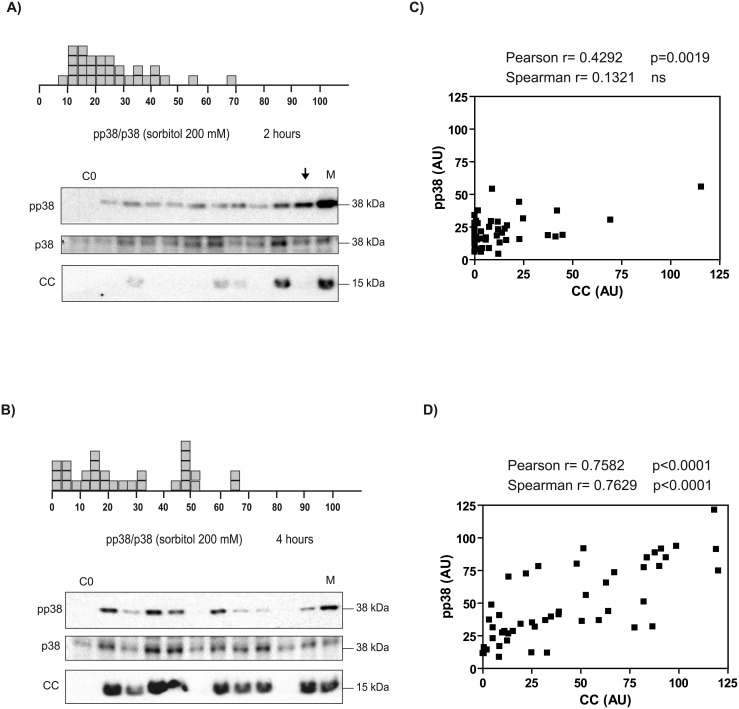
Bimodal and time-dependent response of the p38 system to hyperosmolar sorbitol. (A) Oocytes were incubated with 200 mM sorbitol for 2 h and pp38, p38, and cytochrome c (CC) were measured by Western blot in individual oocytes. p38 activity is represented as pp38/p38 ratio, taking as maximum activity (100% value) the oocytes treated with 400 mM sorbitol (M). Each box represents one individual oocyte. Results are the pool of three independent experiments. The Western blot shows a representative experiment. The arrow indicates no correlation between p38 activation and cytochrome c release at 2 h. (B) p38 activity in individual oocytes treated with 200 mM sorbitol for 4 h. Activity was measured by Western blot, as described previously, and the results expressed as pp38/p38, taking as maximum activity the value obtained with 400 mM sorbitol. (C) Correlation between pp38 and cytochrome c in oocytes incubated with 200 mM sorbitol for 2 h. pp38 and cytochrome c (CC) levels were determined by Western blot in individual oocytes (n = 50) from 5 independent experiments, giving 100% value to individual oocytes treated with 400 mM sorbitol. The data obtained for each oocyte was represented graphically and the correlation coefficient (Pearson r, Spearman r) calculated with GraphPad Prism4. (D) Correlation between pp38 and cytochrome c in oocytes incubated with 200 mM sorbitol for 4 h. pp38 and cytochrome c (CC) were determined by Western blot in individual oocytes (n = 50) as reported before, the data obtained represented graphically, and the correlation coefficients calculated with GraphPad Prism4.

The ultrasensitive responses observed at the level of a population of oocytes could be due to either individual oocytes exhibiting graded (analog) responses or to individual oocytes with all-or-none (digital) responses, as previously reported for AMPK and JNK [12,17]. Moreover, we have reported that the digital response of AMPK and JNK to hyperosmotic shock is time dependent (gradual at 2 h, but digital at 4 h) [12]. Thus, we examined the single cell response of the p38 system to hyperosmolar sorbitol at different times. As we show in Fig 4A, incubation with an intermediate concentration of sorbitol (200 mM) for 2 h induced a homogenous response of p38 in the individual oocytes, with a peak distribution around 20% activity and an extended tail to the right, considering 100% activity the single oocytes treated with sorbitol 400 mM for 4 h. Incubation of the oocytes at an intermediate concentration of sorbitol (200 mM) for 4 h produced a bimodal distribution of p38 activity, with two peaks distributed around 15% and 50% (Fig 4B). Some variability for total p38 was observed in the Western blots due to the stripping of the membranes, which is necessary when analyzing individual oocytes. Importantly, we observed a very good correlation between p38 activation and cytochrome c release at 4 h after treatment (Fig 4B), but not at 2 h (Fig 4A). We quantified by densitometry the pp38 and the corresponding cytochrome c (CC) levels in single oocytes at 2 h (n = 50) and at 4 h (n = 50) and represented graphically the results. A higher correlation coefficient was obtained in the oocytes treated for 4 h (Pearson r = 0.7582, p<0.001) compared with the oocytes treated for 2 h (Pearson r = 0.4292, p = 0.0019). When we calculated the Spearman correlation coefficient, that does not assume a Gaussian distribution of the population, the correlation coefficient at 4 h was r = 0.7629, p<0.0001, and at 2 h was r = 0.1321 (non significant). Note that some oocytes treated with sorbitol for 2 h presented a small amount of cytochrome c release and other oocytes with high pp38 levels did not show cytochrome c release (Fig 2A, arrow). Recently we have reported that other factors (JNK, calpains, and Smac/DIABLO release), in addition to p38 activation, also regulate the early release of cytochrome c induced by osmostress [14].

### Cytochrome c release activates p38, creating a positive feedback loop

**Fig 5:**
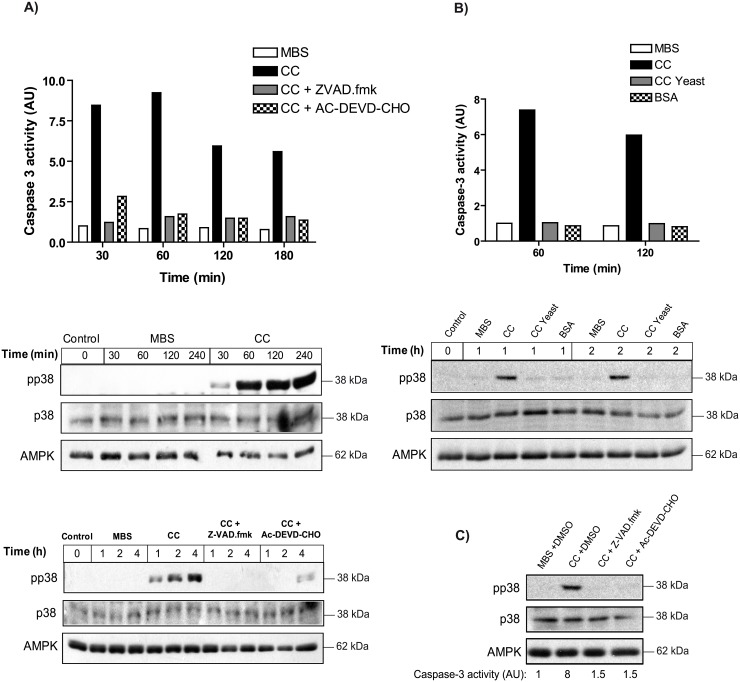
Microinjection of cytochrome c (CC) induces caspase-3 activation and p38 phosphorylation. (A) Caspase inhibitors reduce p38 phosphorylation induced by cytochrome c injection. Oocytes were injected with MBS, horse cytochrome c (CC) (0.5 μM final concentration), CC (0.5 μM) plus Z-VAD.fmk (5 μM), CC (0.5 μM) plus Ac-DEVD-CHO (100 nM), or non injected (control). Pools of 20 oocytes were collected at different times and caspase-3 activity was determined as the concentration of fluorescent AMC formation from Z-DEVD-AMC substrate, and represented as arbitrary units of caspase-3 activity, giving value 1 to MBS injected oocytes. pp38, p38 and AMPK (loading control) were analyzed by Western blot. The result presented is representative of three independent experiments. (B) Injection of BSA or cytochrome c from Saccharomyces cerevisiae (CC Yeast) does not induce caspase-3 activation and p38 phosphorylation. Oocytes were injected with MBS, horse cytochrome c (CC) (0.5 μM), yeast cytochrome c (CC Yeast) (0.5 μM), BSA (0.5 μM), or non injected (control). Pools of 20 oocytes were collected at different times and caspase-3 activity was determined as described before. pp38, p38 and AMPK were analyzed by Western blot. (C) DMSO injection does not interfere with p38 phosphorylation and caspase-3 activation induced by cytochrome c. Oocytes were injected with MBS plus DMSO (diluted 1:50 in MBS), horse cytochrome c (CC) (0.5 μM) plus DMSO (diluted 1:50 in CC), CC (0.5 μM) plus Z-VAD.fmk (50 μM), or CC (0.5 μM) plus Ac-DEVD-CHO (1 μM). Pools of 20 oocytes were collected at different times and caspase-3 activity was determined as described before. pp38, p38 and AMPK were analyzed by Western blot.

**Fig 6:**
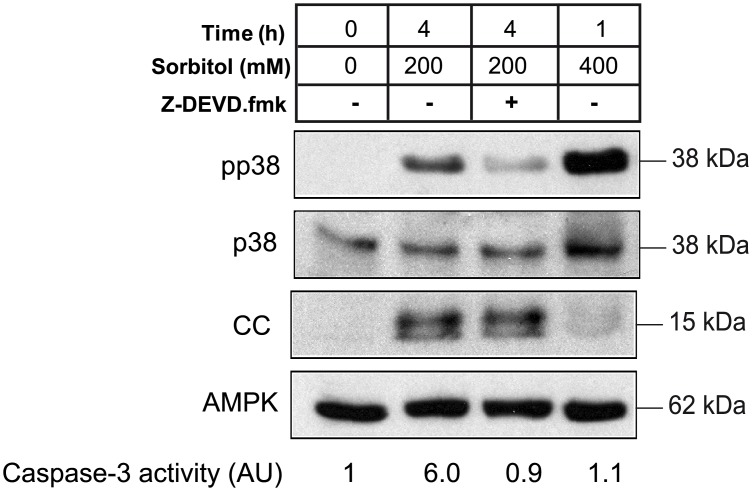
Caspase-3 inhibitor reduces p38 phosphorylation induced by hyperosmotic shock. Oocytes were incubated with 200 mM sorbitol for 4 h in the presence or absence of caspase-3 inhibitor Z-DEVD.fmk (50 μM), or with 400 mM sorbitol for 1 h. Pools of 20 oocytes were collected and pp38, p38, cytochrome c (CC) and AMPK (loading control) were analyzed by Western blot. Caspase-3 activity was determined as the concentration of fluorescent AMC formation from Z-DEVD-AMC substrate, and represented as arbitrary units of caspase-3 activity, giving value 1 to non treated oocytes.

The good correlation obtained between p38 phosphorylation and cytochrome c release in the oocytes exposed to osmostress for 4 h suggests that p38 activation regulates cytochrome c release. Indeed, we have shown that sustained activation of p38 accelerates osmostress-induced apoptosis [14]. However, it is also possible that cytochrome c release might regulate p38 activation, thus creating a positive feedback loop. To address this issue we microinjected horse cytochrome c in *Xenopus* oocytes, which has been reported to induce caspase-3 activation very quickly [18]. As shown in Fig 5A, microinjection of cytochrome c induced caspase-3 activity at 30 min and high p38 phosphorylation levels at 1 h, which was blocked by the general caspase inhibitor Z-VAD.fmk or by the specific caspase-3 inhibitor Ac-DEVD-CHO, demonstrating that p38 activation induced by cytochrome c is caspase-3 dependent. To rule out any effect of proteotoxic stress on p38 activation, bovine serum albumin (BSA) or cytochrome c from Saccharomyces cerevisiae (CC Yeast), which is unable to induce caspase-3 activation in *Xenopus* oocytes [18], were injected at the same concentration that horse cytochrome c. As shown in Fig 5B, injection of horse cytochrome c induced caspase-3 activation and p38 phosphorylation whereas yeast CC or BSA did not. Since caspase inhibitors are dissolved in DMSO, we microinjected DMSO at the same concentration to rule out any effect of the solvent in p38 phosphorylation. As shown in Fig 5C, injection of DMSO diluted 1:50 in MBS (at the same concentration that DMSO in the inhibitors) did not induce p38 phosphorylation in the oocytes and did not interfere with the phosphorylation induced by horse cytochrome c, whereas the inhibitors Z-VAD.fmk or Ac-DEVD-CHO completely blocked p38 phosphorylation and caspase-3 activity. These results suggest that hyperosmotic shock induces a rapid activation of the p38 signaling pathway (15 min to 2 h, independent of caspase-3) and a late activation (2–4 h) triggered by cytochrome c release and caspase-3 activation, thus accounting for the good correlation observed at 4 h (Fig 4B and 4D). The engagement of this feedback loop could also explain the residual levels of p38 activity detected in oocytes washed and returned to normal medium (Fig 3). When *Xenopus* oocytes were exposed to 200 mM sorbitol for 4 h in the presence or absence of the caspase-3 inhibitor Z-DEVD.fmk, cytochrome c was released at similar levels in both conditions, whereas p38 phosphorylation was reduced in the oocytes incubated with the inhibitor (Fig 6). These results indicate that caspase-3 activation induced by hyperosmotic shock regulates p38 activation. As shown in Fig 6, p38 was activated in oocytes exposed to 400 mM sorbitol for 1h without cytochrome c release and caspase-3 activation. Therefore, hyperosmotic shock induces an early activation of p38 independently of cytochrome c release and caspase-3 activation, in contrast to the late activation of p38.

## Discussion

This study shows, for the first time, four important properties of the p38 signaling system in response to hyperosmotic shock.

First, p38 signaling pathway is highly ultrasensitive (n_H_≈14.4) to hyperosmolar stress. This allows rapid activation of the sensor after a certain threshold level is surpassed, and emphasizes the importance of the p38 pathway as a primary response to osmostress. The response is even higher that for JNK (n_H_≈ 8.8) or AMPK (n_H_≈ 5.3) signaling pathways, which we had previously reported [12]. It is remarkable that in *Xenopus* oocytes there is not transcriptional activity and, therefore, all biological effects as a consequence of p38 activation must be genomic independent. It has been reported that nuclear translocation of Hog1 (the homologous of p38 in yeast) and the effects on transcription that it normally causes are not necessary for its role in hyperosmotic stress resistance. By contrast, enzymes needed for glycerol production were essential for maintenance of osmotic balance and viability [19]. It would be interesting to study if there is any metabolic pathway regulated by p38 activation that could increase the intracellular concentration of an osmoprotectant in *Xenopus* oocytes.

Second, p38 signaling system is monostable, meaning that when the stimuli disappears the activity returns to basal levels. This is similar to the AMPK signaling system, and in contrast with the observed bistability of the JNK system, as we previously reported [12]. We think that this differential behaviour of protein kinases versus the same stress might be important for the adaptation of the cell to osmotic shock and for regulation of the cell death program. The combination of monostable and bistable systems may be useful to evaluate the strength and duration of a noxious stimulus and to establish a point of no return when a certain threshold level has been surpassed [13]. Although we consider that p38 signaling system is monostable, some residual activity is present in the oocytes exposed and returned to normal medium (Fig 3), as a consequence of cytochrome c release induced by osmostress. The magnitude and duration of the p38 signal might be important for its biological effects. Downregulation of signaling is required in yeast; as sustained activation of Hog1 is detrimental to cell growth [20]. Indeed, sustained activation of p38 accelerates osmostress-induced apoptosis in *Xenopus* oocytes [14]. In many cell lines, and under certain continuous stimuli, the activation of p38 is transient, indicating the existence of down-regulation mechanisms, including activation of different phosphatases [1,2]. For instance, the activity of MAPKs can be regulated by a family of DUSPs (dual-specificity phosphatases), which are transcriptionally up-regulated by stimuli that activate MAPK signaling, and are thought to play an important role limiting the extent of MAPK activation [21,22]. In *Xenopus* oocytes the transcriptional effects are not possible and this might explain why p38 activation is persistent when the stimulus is continuous (Fig 1) in contrast with other cellular systems [23,24].

Third, by single cell analysis we found that the response of p38 to hyperosmolar sorbitol is bimodal, in contrast to the all-or-none response previously reported for AMPK and JNK [12]. However, a common feature of ultrasensitive kinases in front to hyperosmotic shock is the generation of two different populations of kinase activity from an initial homogenous response. The presence or absence of feedback loops for specific signalling pathways might explain a bimodal or an all-or-none distribution. Recently, it has been described that Hog1 gradually accumulates in the nucleus after increasing salt concentration, but the transcriptional output obtained is bimodal [25]. The authors did not measure p38 phosphorylation levels, but assume that Hog1 nuclear accumulation is linked to its kinase activity. However, we measured p38 phosphorylation, which is well correlated with p38 activity, obtaining a bimodal distribution at 4 h after osmostress. The different methodology used can explain the distinct results obtained in both studies. Alternatively, the differential response to hyperosmotic stress observed in *Xenopus* and *Saccharomyces* could be due to the lack of transcripcional activity or to the induction of apoptosis in *Xenopus* oocytes. Evidences from several studies indicate that p38 MAPK pathway has a role in cellular differentiation [1,2], or regulates checkpoint controls and cell cycle transitions [26]. It would be interesting to investigate whether p38 shows a bimodal response to other stimuli. This might not be a general rule since, as we have reported, AMPK shows a digital response to osmotic stress but a graded response to antimycin [12].

Fourth, high levels of cytochrome c in the cytosol induces p38 phosphorylation through caspase-3 activation. Therefore, our results imply that sustained activation of p38 induced by hyperosmotic shock accelerates cytochrome c release and caspase-3 activation [14], which in turn activates p38 thus creating a positive feedback loop. This positive feedback loop, in combination with others engaged by caspase-3 [27,28], could be important to make irreversible the apoptotic program. High levels of cytochrome c, released from the mitochondria, would be a point of no return. Importantly, this feedback loop might explain the bimodal distribution observed at 4 h after osmostress and the residual p38 activity in the oocytes washed and incubated in normal medium. The marked reduction of p38 activity when oocytes are washed suggests that late p38 activation induced by caspase-3 involves a different signaling pathway that early p38 activation induced by hyperosmotic shock. It has been reported that caspase-3 induces proteolysis and constitutive activation of MEKK1 [29,30], which in turn activates JNK and p38 [31]. However, cytochrome c injection did not induce a rapid phosphorylation of JNK (data not shown), thus discarding proteolysis and constitutive activation of MEKK1. Another possibility is that caspase-3 activation would increase the levels of reactive oxygen species (ROS) through disruption of the functions of complex I and II of the electron transport chain [32], which in turn would activate p38 through activation of MINK and/or ASK1 [33,34]. More studies are necessary to characterize the signaling pathway that induces p38 phosphorylation through caspase-3 activation.

Hyperosmotic stress occurs in diverse pathological conditions such as diabetes mellitus, heat shock, infections, and dehydration after exercise, affecting different tissues [35–38]. Therefore, to know the signaling properties of the p38 pathway in response to hyperosmotic shock can be useful to design computational models to predict cellular responses in these pathological conditions.

## Conclusions

p38 is highly ultrasensitive in response to hyperosmotic shock. At a single cell level, p38 shows an initial gradual response to osmostress at 2 h, which is converted into a bimodal response at 4 h. The good correlation between cytochrome c release and p38 activation at 4 h after hyperosmotic shock is due to: (1) sustained activation of p38 promoting cytochrome c release from the mitochondria and, (2) a positive feedback loop engaged after cytochrome c release and caspase-3 activation, which in turn induces p38 phosphorylation. These properties give insight into the mechanisms that regulate osmostress-induced apoptosis and facilitate the design of computational models to predict the response of protein kinases during the cell death program.

## Supporting Information

S1 ARRIVE Checklist NC3Rs ARRIVE Guideline Checklist 2014. (DOCX)
