# Supplementary material for: Basic Properties of the p38 Signaling Pathway in Response to Hyperosmotic Shock
Source: PLoS One. 2015 Sep 3;10(9):e0135249. doi: 10.1371/journal.pone.0135249 (PMC4559375; doi:10.1371/journal.pone.0135249)

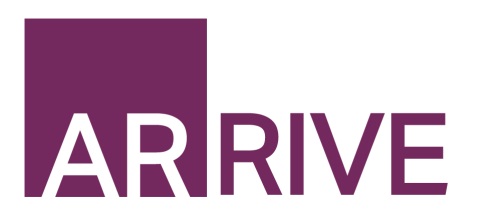


The ARRIVE Guidelines Checklist

Animal Research: Reporting In Vivo Experiments

Carol Kilkenny^1^, William J Browne^2^, Innes C Cuthill^3^, Michael Emerson^4^ and Douglas G Altman^5^

*^1^The National Centre for the Replacement, Refinement and Reduction of Animals in Research, London, UK, ^2^School of Veterinary Science, University of Bristol, Bristol, UK, ^3^School of Biological Sciences, University of Bristol, Bristol, UK, ^4^National Heart and Lung Institute, Imperial College London, UK, ^5^Centre for Statistics in Medicine, University of Oxford, Oxford, UK.*

|  | | ITEM | RECOMMENDATION | Section/ Paragraph |
| --- | --- | --- | --- | --- |
| 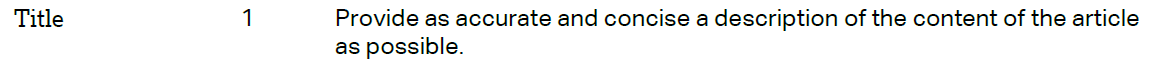 | | | Title |  |
| 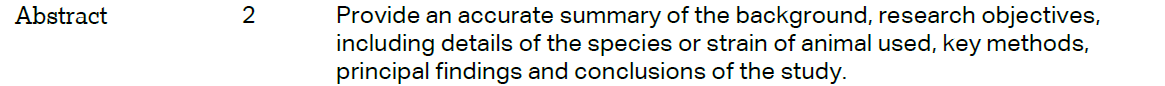 | | | Abstract |  |
| INTRODUCTION | | |  |  |
| 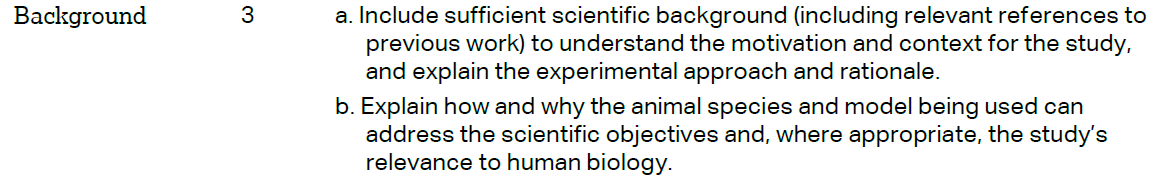 | | | Page 3-4, paragraphs 3-4.  Page 3-4, paragraphs 3-4. |  |
| 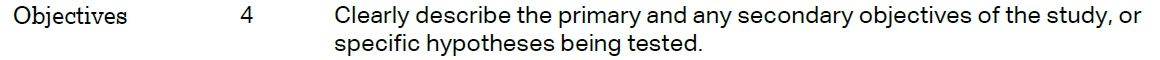 | | | Page 3-4, paragraph 3. |  |
| METHODS | | |  |  |
| 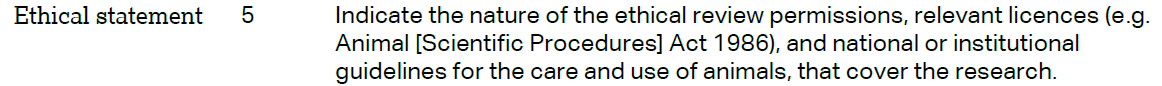 | | | Page 4, paragraph 1. |  |
| 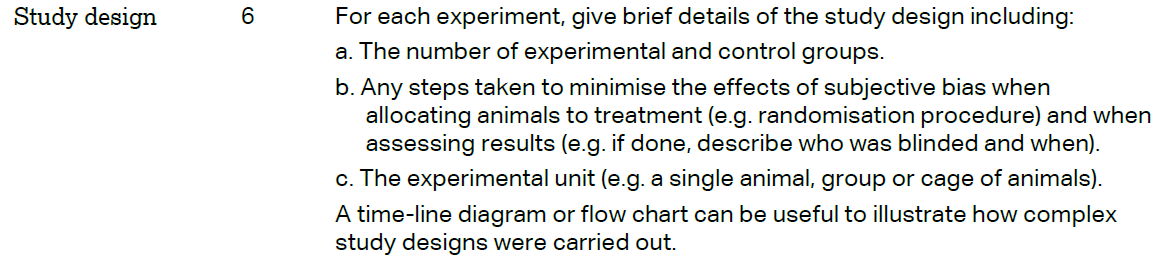 | | | Page 17-18, figure legends 1-6.  Page 4, paragraph 1. |  |
| 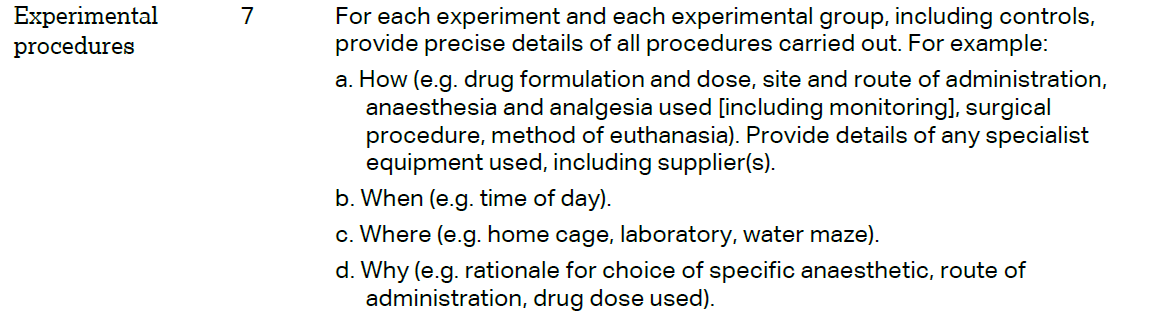 | | | Page 4, paragraph 1. |  |
| 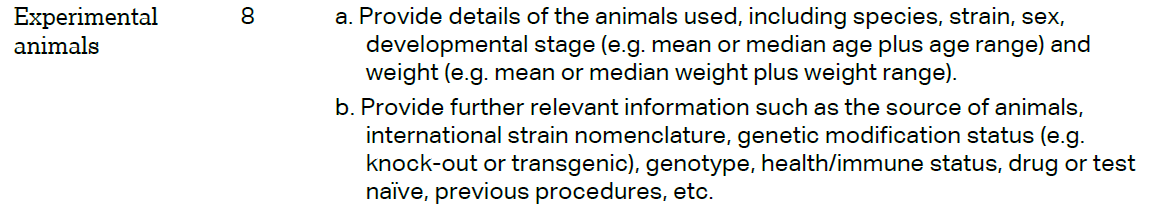 | | | Page 4, paragraph 1.  Page 4, paragraph 1. |  |

The ARRIVE guidelines. Originally published in *PLoS Biology*, June 2010^1^

| 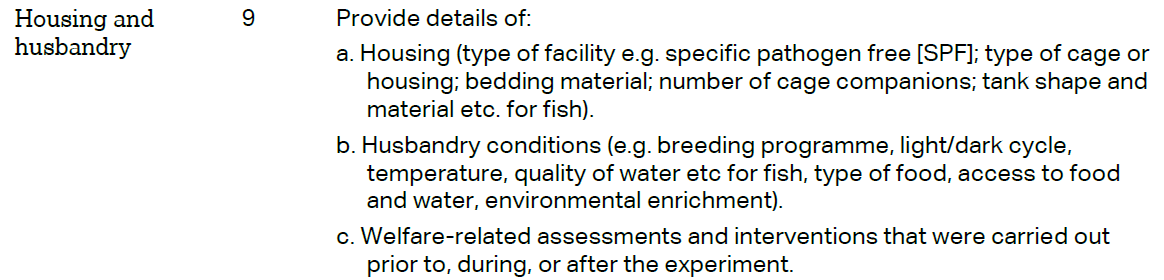 | Page 4, paragraph 1. | |
| --- | --- | --- |
| 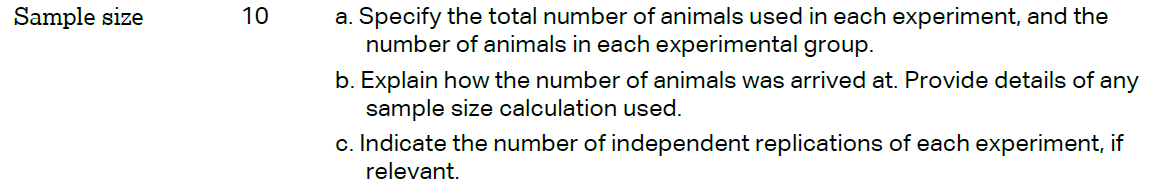 | Page 17-18, figure legends 1-6. | |
| 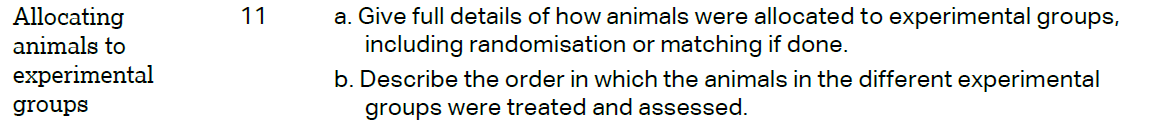 | Page 4, paragraph 1. | |
| 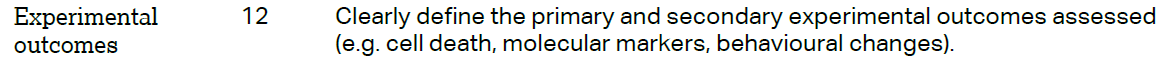 | Page 4, paragraph 1. | |
| 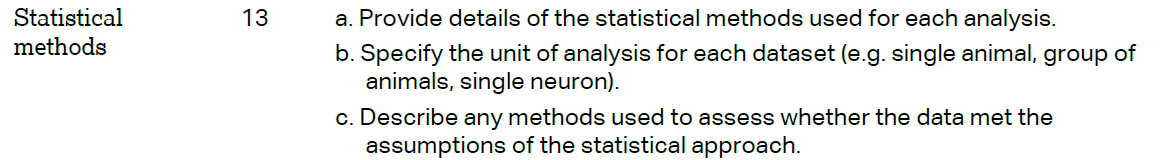 | Page 17-18, figure legends 1-6. | |
| RESULTS |  | |
| 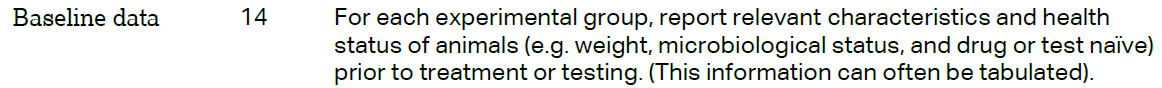 | Frogs were not treated. Only the oocytes obtained from frogs were treated. | |
| 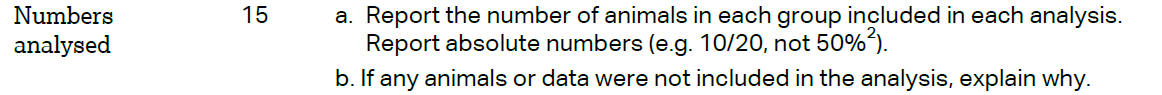 | Non aply (see item 14). | |
| 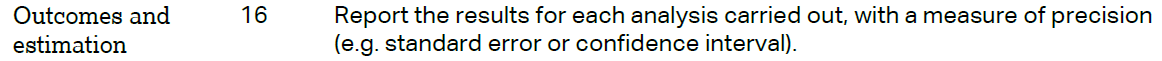 | Pages 6-7, paragraphs 1-2. Figures 1-4 | |
| 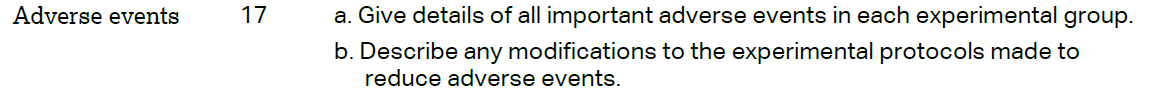 | Non aply (see item 14). | |
| DISCUSSION |  | |
| 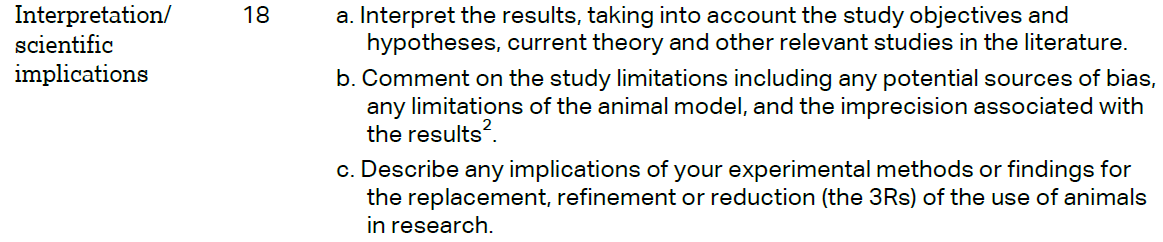 | Pages 8-11  Page 9, paragraph 1. Page 10, paragraph 1. | |
| 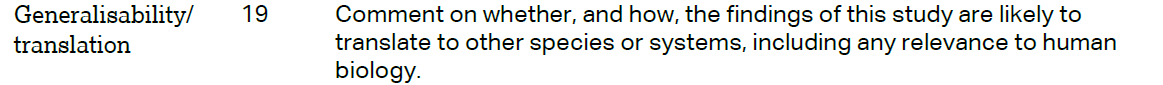 | Page 11, paragraphs 2-3. | |
| 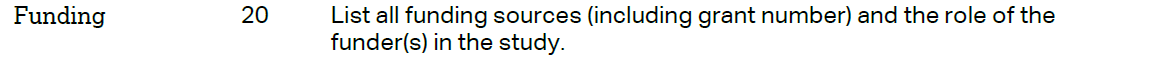 | | Funding included in the on line submission (page 1). |


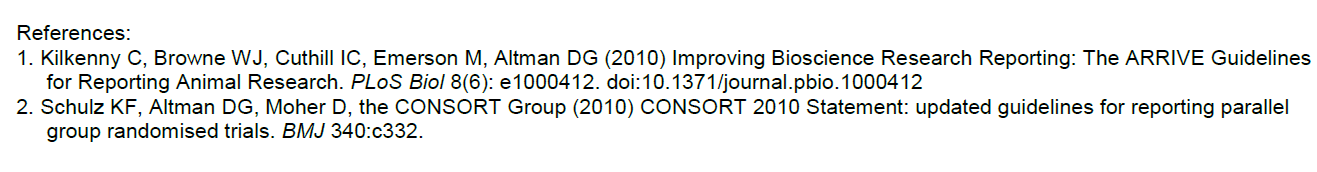

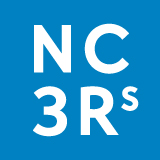

Supplement: S1 ARRIVE Checklist — (DOCX) [file pone.0135249.s001.docx]
